# Supplementary material for: DNA evidence of bowhead whale exploitation by Greenlandic Paleo-Inuit 4,000 years ago
Source: Nat Commun. 2016 Nov 8;7:13389. doi: 10.1038/ncomms13389 (PMC5105157; doi:10.1038/ncomms13389)
Supplement: Supplementary Information — Supplementary Figures 1-5, Supplementary Tables 1-12, Supplementary Notes 1-3, Supplementary Methods and Supplementary References. [file ncomms13389-s1.pdf]

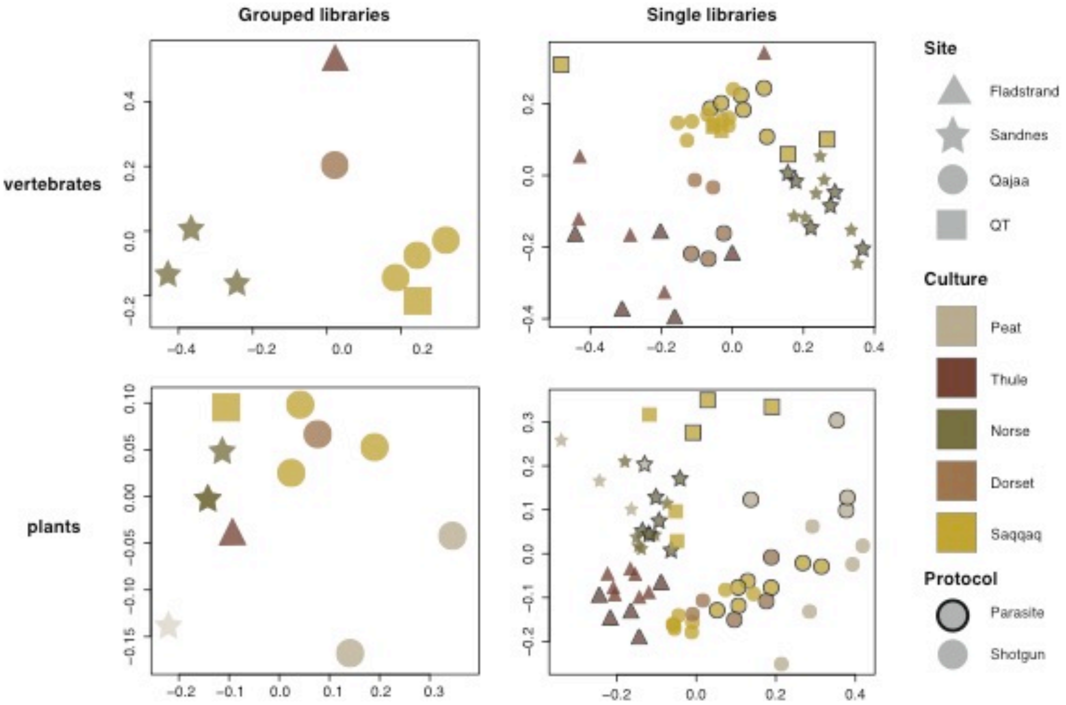

Supplementary Figure 1 | Variation within grouped and single libraries based on DNA reads assigned to vertebrates and plants. For vertebrate DNA, distance calculations are based on the 42 vertebrate taxa identified in Supplementary Table 2 and 3. Due to absence of vertebrate DNA, peat layers were not included. Distance calculations for plants were based on plant families represented by more than 50 reads across the entire data set. Grouped libraries represents several single libraries merged into groups according to the sections presented in Figure 1.

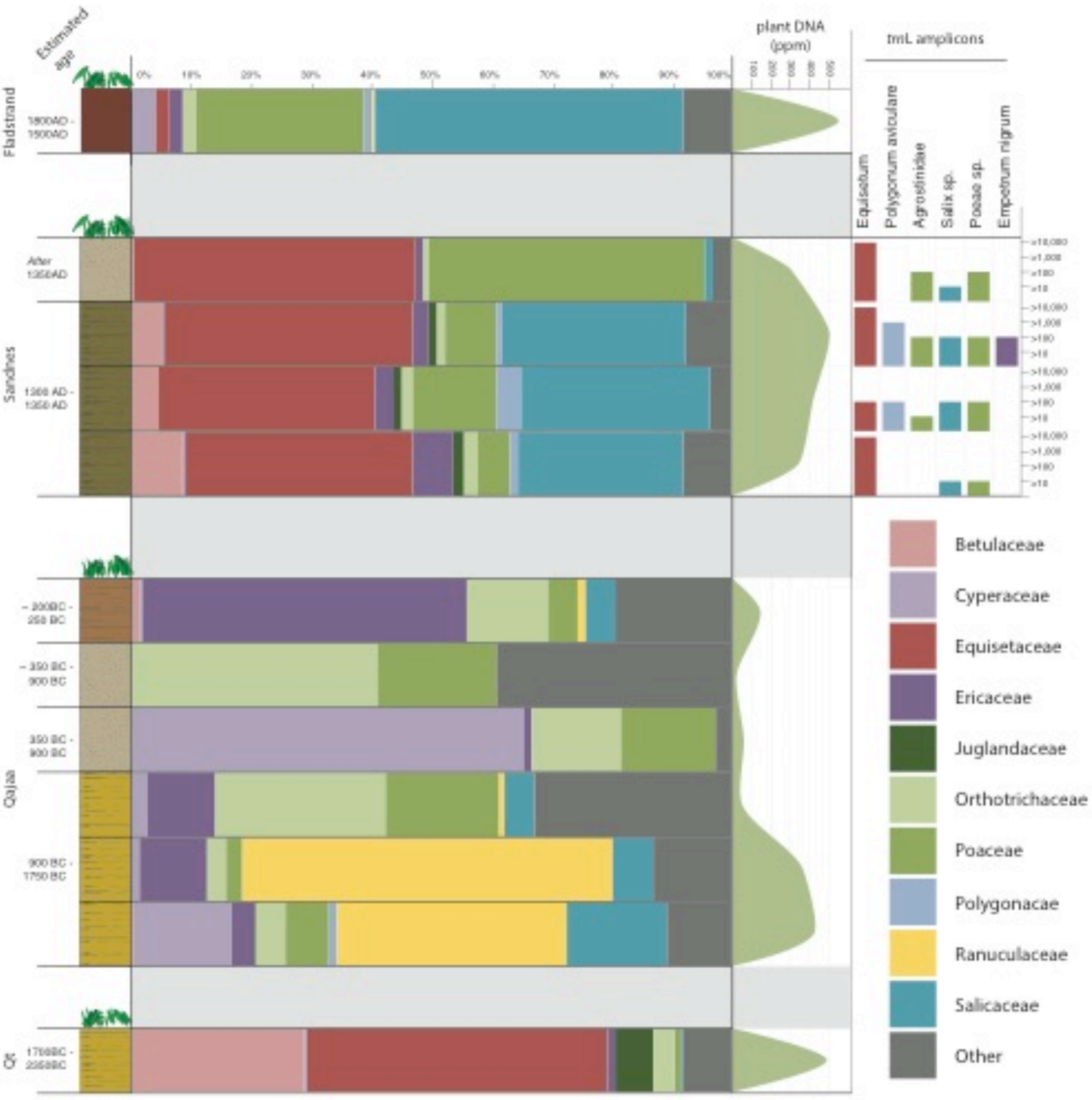

Supplementary Figure 2 | Plant abundance. Relative abundance of the 10 most common plant families, based on read counts in Supplementary Table 5. Plant DNA concentration is defined as plant DNA reads pr million reads analyzed. *trn*L amplicons represents the 6 most abundant taxa identified. For each layer, only taxa identified in 2 or more replicates were included.

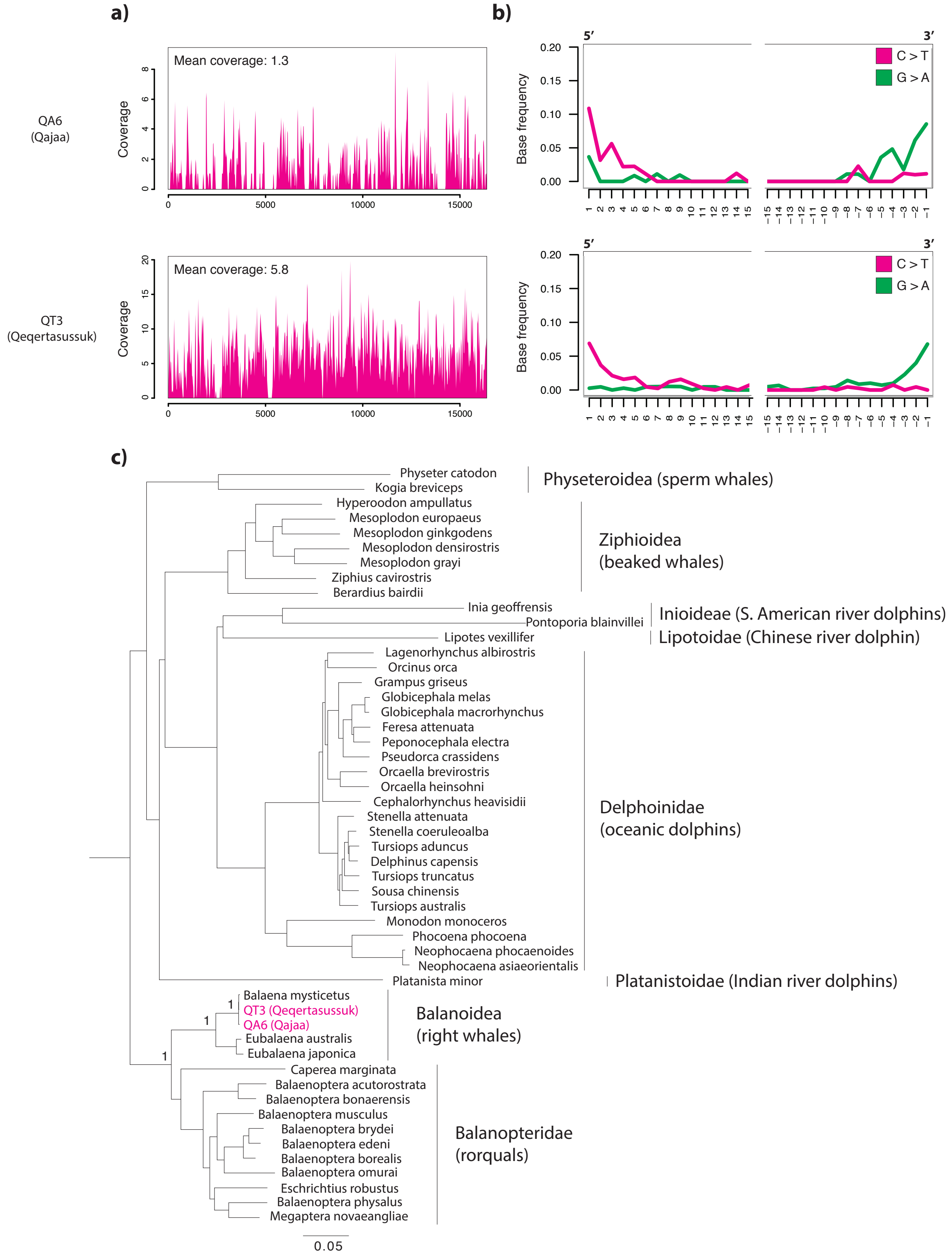

Supplementary Figure 3 | Recovery of bowhead whale mitochondrial genomes from library QA6 at Qajaa and QT3 at Qeqertasussuk. The mitochondrial genomes are based on reads assigned below family level, with the *Balaena mysticetus* reference genome among the best hits. **a)** Coverage plot of reads mapped to the bowhead whale reference mitochondrial genome (gi: 38707506). Green line presents the average depth of coverage. **b)** Nucleotide misincorporation pattern displaying the DNA damage pattern for the aligned reads. **c)** Bayesian tree illustrating the location of the recovered bowhead whale mitochondrial genomes (pink labels) in the phylogenetic tree of whales. The consensus tree was constructed, using hippopotamus (*Hippopotamus amphibius*, gi: 5836030) as an out-group (not shown). Relevant posterior probabilities are shown.

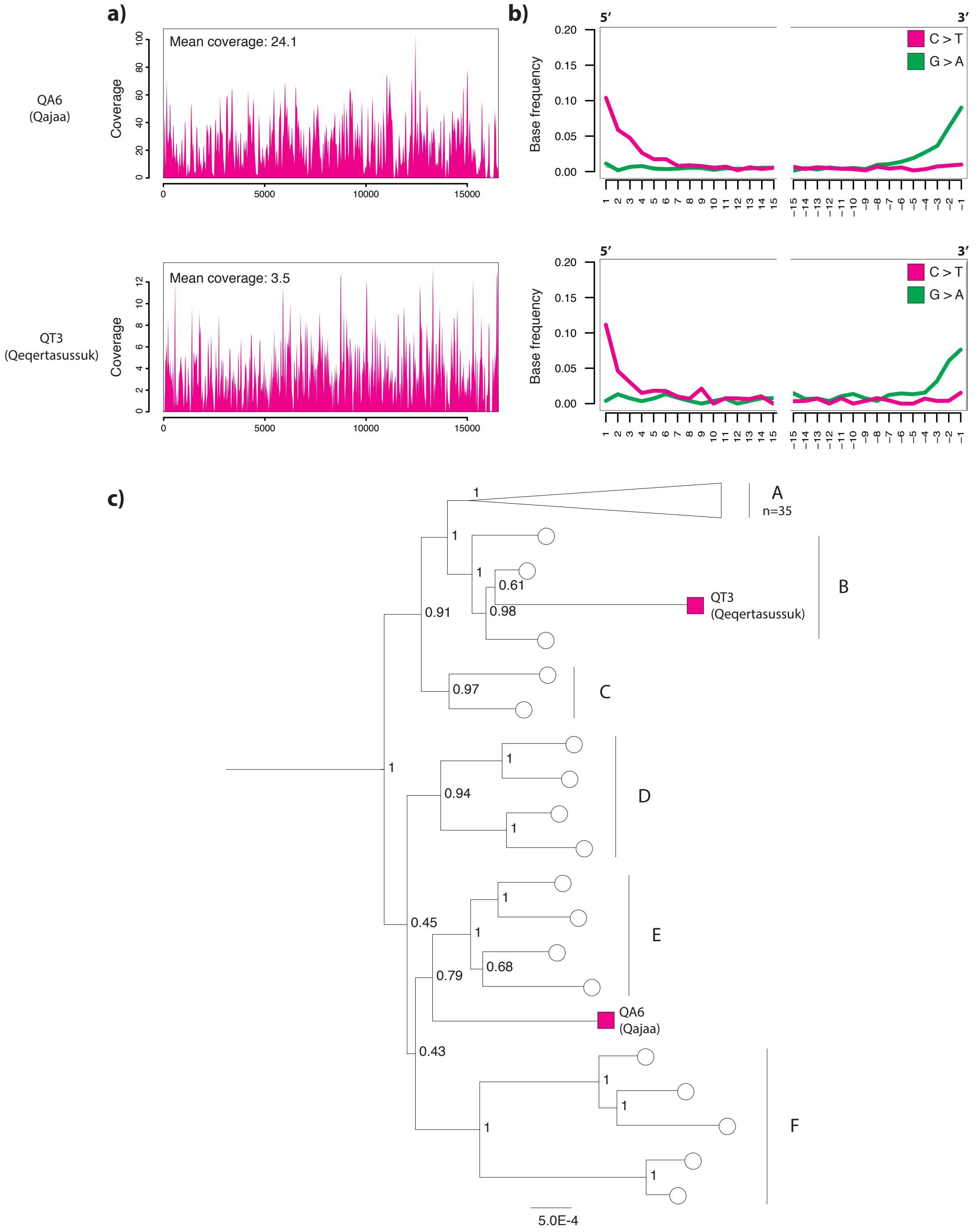

Supplementary Figure 4 | Recovery of harp seal mitochondrial genomes from library QA6 at Qajaa and QT3 at Qeqertasussuk. The mitochondrial genomes are based on reads assigned below family level, with the *Pagophilus groenlandicus* reference genome among the best hits. **a)** Coverage plot of reads mapped to the harp seal reference mitochondrial genome (gi:115494733). Green line presents the average depth of coverage. **b)** Nucleotide misincorporation pattern displaying the DNA damage pattern for the aligned reads. **c)** Bayesian tree illustrating the relationship between the recovered harp seal mitochondrial genomes (pink squares) and 6 harp seal haplogroups (A, B, C, D, E, F) from Carr et al. 2015 (open circles)[23]. The subtree representing haplo group A has been collapsed for clarity. The consensus tree was constructed, based on 54 harp seal mitochondrial genomes, using ribbon seal (*Phoca fasciata*, gi:115494719) as an out-group (not shown). Node labels represent posterior probabilities.

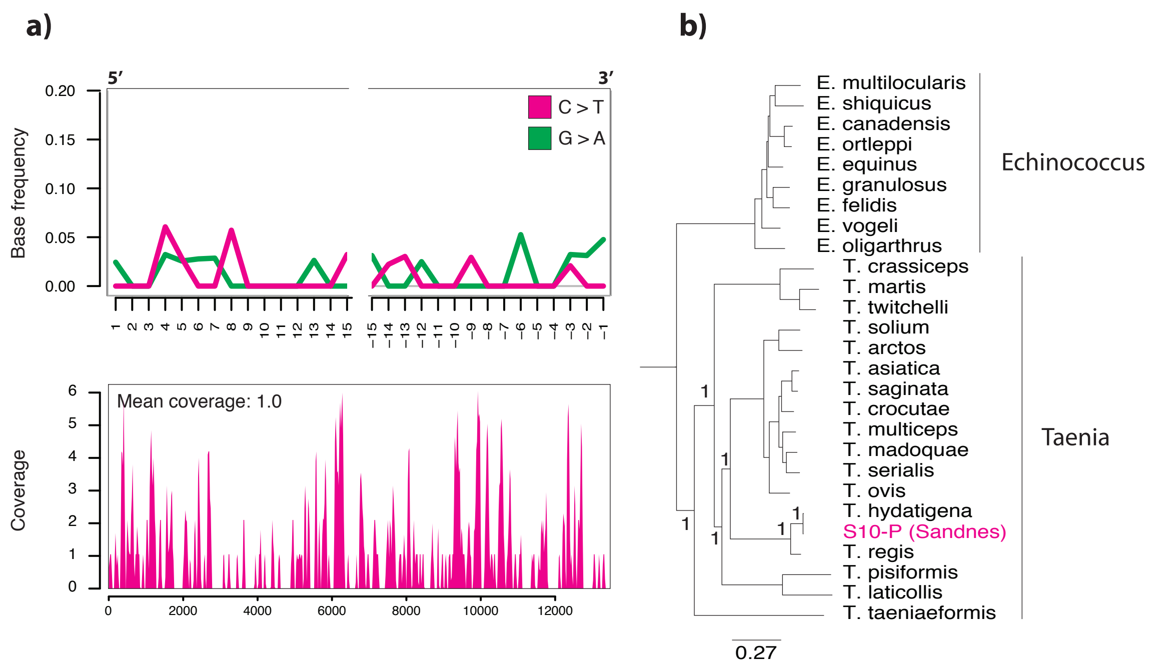

Supplementary Figure 5 | Recovery of a *Taenia hydatigena* mitochondrial genomes from library S10-P at Sandnes. The mitochondrial genome is based on reads assigned below family level, with the *Taenia hydatigena* (gi: 242613260) reference genome among the best hits. **a)** Nucleotide misincorporation pattern for the aligned reads. **b)** Coverage plot of reads mapped to the *Taenia hydatigena* reference mitochondrial genome. Green line represents the average depth of coverage **c)** Bayesian tree illustrating the location of the recovered mitochondrial genome (pink identifier) in the phylogenetic tree of tapeworms. The consensus tree was constructed, using *Hymenolepis diminuta* (gi:14018028) as an outgroup (not shown). Relevant posterior probabilities are shown.

taxa	Fladstrand	Norse A	Norse B	Norse C	Norse D	Dorset	Peat B	Peat A	Late Saqqaq	Middle Saqqaq	Early Saqqaq	QT
Bovidae	-	-	15	12	11	-	-	-	-	-	-	3*
Bovinae	-	-	16	12	12	-	-	-	-	-	-	-
Bos	2*	-	74	70	105	5*	3*	3*	-	-	-	3*
Caprinae	-	-	12	5	-	-	-	-	-	-	-	-
Capra	-	-	36	13	17	-	-	-	-	-	-	-
Ovis	-	-	23	9	15	-	-	-	-	-	-	-
Canis	29	-	2*	3*	4*	3	-	-	-	-	-	-
Canis lupus	41	-	-	6*	-	19	-	-	-	6	-	-
Canis lupus familiaris	21	-	-	-	-	7	-	-	-	2	-	-
Phocidae	-	-	-	7	13	46	-	-	21	1026	58	787
Pagophilus groenlandicus	4	-	6	9	28	9	-	-	85	6357	303	1041
Erignathus barbatus	-	-	2	-	-	-	-	-	4	62	23	6
Cystophora cristata	-	-	-	-	-	-	-	-	2	-	-	18
Phoca	-	-	-	2	3	-	-	-	9	361	17	98
Phoca vitulina	-	-	-	-	8	-	-	-	-	6	6	35
Pusa	-	-	-	-	-	14	-	-	-	-	3	82
Pusa hispida	3	-	-	-	-	41	-	-	-	-	8	197
Balaenidae	-	-	-	-	-	2	-	-	9	53	24	360
Balaena mysticetus	-	-	-	-	-	5	-	-	32	339	107	1736
Eubalaena	-	-	-	-	-	-	-	-	-	2	4	23
Monodon monoceros	7	-	-	-	-	-	-	-	2	69	-	-
Odobenus rosmarus	-	-	4	3	2	-	-	-	2	89	35	-
Cervidae	-	-	-	-	-	-	-	-	-	55	-	71
Odocoileinae	-	-	2	-	-	-	-	-	-	30	-	82
Rangifer tarandus	2	-	-	-	21	-	-	-	4	567	7	657
Lepus	11	-	-	-	-	-	-	-	-	-	-	-
Phoca largha(?)	-	-	-	-	-	2	-	-	-	5	-	10
Pusa sibirica (?)	-	-	-	-	-	4	-	-	-	-	-	10
Pusa caspica (?)	-	-	-	-	-	-	-	-	-	-	-	20
Phoca fasciata (?)	-	-	-	-	-	2	-	-	-	7	-	8

Supplementary Table 1 | Mammal read counts. Read counts of mammal taxa represented by more than 10 reads in the dataset. Read counts below two are not shown. (?) Presumed false positives. * Presumed contamination

taxa	Flad-strand	Norse A	Norse B	Norse C	Norse D	Dorset	Peat B	Peat A	Late Saqqaq	Middle Saqqaq	Early Saqqaq	QT
Laridae	-	-	-	-	-	2	-	-	-	17	19	5
Larus	-	-	-	-	-	-	-	-	-	20	20	3
Larus dominicanus (+)	-	-	-	-	-	-	-	-	2	39	43	-
Anatidae	7	-	-	-	-	-	-	-	2	12	10	4
Branta	9	-	-	-	-	-	-	-	-	-	5	-
Branta canadensis	27	-	-	-	-	-	-	-	-	9	29	-
Gonorynchus greyi (+)	5	-	-	-	-	-	-	2	-	10	3	4
Pterothrissus gissu (+)	4	-	-	-	-	-	-	-	2	-	2	5
Notropis stramineus (+)	-	-	-	-	2	-	-	-	4	6	-	-
Hypomesus nipponensis (+)	-	-	2	-	6	-	2	-	3	3	2	2
Lates calcarifer (+)	-	-	-	-	-	-	-	-	3	-	6	3
Turdus merula (?)	13	-	4	2	4	2	5	-	9	2	5	28

Supplementary Table 2 | Other vertebrate read counts Read counts of non-mammal taxa represented by more than 10 reads in the dataset. Read counts below two are not shown. (?) Presumed false positives. (+) Presumed closest match assignment.

taxa	Flad-strand	Norse A	Norse B	Norse C	Norse D	Dorset	Peat B	Peat A	Late Saqqaq	Middle Saqqaq	Early Saqqaq	QT
Taenia crocutae	-	-	-	-	3	-	-	-	-	-	-	-
Taenia multiceps	6	-	2	3	17	-	-	-	-	-	-	-
Taenia	6	-	6	11	38	-	-	-	-	-	-	-
Taenia hydatigena	-	-	25	34	285	-	-	-	-	-	-	-
Taenia serialis	-	-	-	-	4	-	-	-	-	-	-	-
Echinococcus canadensis*	-	-	-	-	8	-	-	-	-	-	-	-
Echinococcus	-	-	-	-	5	-	-	-	-	-	-	-
Echinococcus ortleppi	-	-	-	-	2	-	-	-	-	-	-	-
Taenia asiatica	-	-	-	-	2	-	-	-	-	-	-	-
Taenia regis	-	-	-	-	4	-	-	-	-	-	-	-
Taeniidae	-	-	-	-	2	-	-	-	-	-	-	-
Toxocara canis*	-	-	-	-	-	-	-	-	-	-	2	-

Supplementary Table 3 | Parasite read counts. Parasite read counts within the families Taeniidae and Taxocaridae from each group. Read counts below two are not shown. *Species with zoonotic properties.

ID #	56	57	58	60	62	204	209
Site	Sandnes	Sandnes	Sandnes	Sandnes	Sandnes	Fladstrand	Fladstrand
Sample	V51-4	V51-5	V51-6	V51-8	V51-10	Cla6-100.0-203-2	Cla6-100-201.0
Processed for egg recovery (g)	26.8	35.7	40.9	32	25.2	14.3	19.2
Eggs in processed sample	40	90	30	10	10	10	40
Tapeworm eggs isolated per gram sample	1.5	2.5	0.7	0.3	0.4	0.7	2.1
Size of egg(s) (mm)	31 x 27	33 x 30	35 x 31	33 x 31	35 x 30	33 x 30	33 x 29
Egg(s) imaged	4	9	3	1	1	1	4
Representative egg	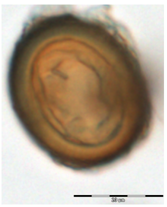	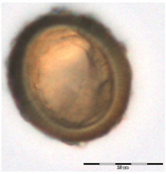	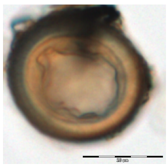	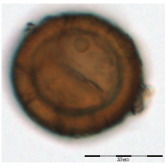	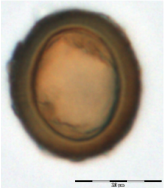	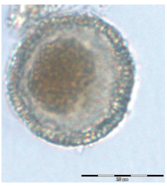	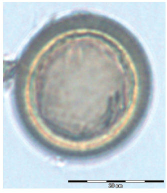

Supplementary Table 4 | Morphological characterization of parasitic eggs.

taxa	Flad-strand	Norse A	Norse B	Norse C	Norse D	Dorset	Peat B	Peat A	Late Saqqaq	Middle Saqqaq	Early Saqqaq	QT
Betulaceae	-	86	1930	995	3219	257	-	-	-	55	471	42334
Cyperaceae	5515	-	147	-	83	111	-	2312	194	1899	17698	102
Equisetaceae	3432	15912	16749	8440	13940	-	-	-	-	-	-	73357
Ericaceae	3221	375	1069	791	2514	10031	-	56	797	16865	4510	1974
Juglandaceae	-	-	445	229	648	54	-	-	-	-	114	8882
Orthotrichaceae	2931	377	698	416	879	2544	1346	518	2038	5062	5334	5524
Poaceae	40193	15452	3300	3307	1946	868	651	557	1358	3935	7834	1102
Polygonaceae	2109	-	408	890	525	55	-	-	-	186	1552	-
Ranunculaceae	153	-	-	-	-	226	-	-	67	92426	42045	175
Salicaceae	73626	564	12390	7410	10157	878	-	-	364	10787	18184	501
Other	11474	1039	3031	839	2980	3668	1294	103	2362	19479	11833	12099

Supplementary Table 5 | Plant read counts. Plant read counts from the ten most common plant families in each group. Counts below 50 are not shown.

taxa	Flade-strand	Norse A	Norse B	Norse C	Norse D	Dorset	Peat B	Peat A	Late saqqaq	Middle saqqaq	Early saqqaq	QT
Betulaceae: Ostrya rehderiana	-	-	2	3	1.1	-	-	-	-	-	6.5	4.2
Cyperaceae: Carex siderosticta	2.2	-	-	-	-	-	-	9.8	-	6.9	8.8	-
Equisetaceae: Equisetum arvense	0.3	1	0.5	0.9	0.6	-	-	-	-	-	-	1.2
Ericaceae: Vaccinium macrocarpon	1.1	-	0.4	0.6	1.5	2.4	-	-	6.6	6.5	8.6	4.3
Juglandaceae: Juglans regia	-	-	-	-	0.6	-	-	-	-	-	-	3.7
Orthotrichaceae: Nyholmiella obtusifolia	1.6	-	1.4	-	1.4	2.3	1.2	5	7.9	8.1	6.3	4.8
Poaceae: Poa palustris	0.9	0.9	1.9	1.9	1.8	-	-	-	9.3	7.2	6.2	-
Polygonaceae: Rheum palmatum	0.6	-	-	2.8	-	-	-	-	-	-	6.6	-
Ranunculaceae: Ranunculus macranthus	-	-	-	-	-	-	-	-	-	7.2	6.5	-
Salicaceae: Salix suchowensis	1	0.9	1.5	1	1	1.5	-	-	-	9.7	9.1	-
Balaena mysticetus	-	-	-	-	-	-	-	-	-	-	-	7.1
Phoca groenlandica	-	-	-	-	-	-	-	-	-	10.7	-	10.6
Rangifer tarandus	-	-	-	-	-	-	-	-	-	8.9	-	13.1

Supplementary Table 6 | DNA damage. DNA damage on the first 5' position, from grouped samples. For each plant family, the most abundant species in each family was chosen as reference.

	Age category	Live weight (kg)	MNI	Biomass (kg)	Abundance (%)
**Arctic fox**		3.00	903	2709	0.9
**Ringed seal**	0-3 yrs	25.00	1764	44100	15.3
	3 - yrs	35.00	352	12320	4.3
**Harp seal**	0-1 yrs	30.00	920	27600	9.5
	1-4 yrs	75.00	922	69150	23.9
	5- yrs	130.00	884	114920	39.7
**Fulmar**		0.75	6630	4972.5	1.7
**Ptarmigan**		0.50	1168	584	0.2
**Gulls**		1.50	1914	2871	1
**Little auk**		0.15	1634	245.1	0.1
**Brunnich's guillemot**		1.10	8769	9645.9	3.3
**Total**			**25860**	**289117.5**	**100**

Supplementary Table 7 | MNI estimates from Qeqertasussuk. The table represents the estimates presented in column 3 (All faunal components) from table 9.5 in Meldgaard 2004.

species	NISP	corr. NISP	sedaDNA
Canis lupus	18	18	8
Arctic hare	1	1	0
Arctic fox	22	22	0
Reindeer	10	10	663
Harbour seal	1	30	12
Ringed seal	119	3609	11
Harp seal	339	10281	6745
Bearded seal	1	30	89
Narwhal	2	2	71
Seagulls	775	856	178
Other birds	263	290	71
Unidentified bird sp.	108	-	-
Unidentified seal sp	13491	-	-

Supplementary Table 8. Comparison of sedaDNA data with the bone record from Qajaa. NISP and corrected NISP data are from[13]. In the corrected NISP counts, higher order taxa (birds sp. and seal sp.) are divided between the represented bird and seal species according to their relative abundances. sedaDNA data represents merged reads from the Saqqaq layers at Qajaa. In cases where higher order taxa could be uniquely identified to a single species, reads were collapsed to species level. Pearson's rho=0.93, p=3.2e-5.

Library ID	Group	pp reads	Sample type	CGG ID	Sediment ID
FA1	Fladstrand	5668844	sediment	CGG-3-006350	Cla6-100.0-203-3
FA2-P	Fladstrand	5718668	parasite eggs	CGG-3-006349	Cla6-100.0-203-2
FA2	Fladstrand	82275125	sediment	CGG-3-006349	Cla6-100.0-203-1
FA3	Fladstrand	28605815	sediment	CGG-3-006348	Cla6-100.0-203-1
FB1-P	Fladstrand	6920566	parasite eggs	CGG-3-006351	Cla6-104.25-209.25-1
FB2	Fladstrand	30681091	sediment	CGG-3-006352	Cla6-104.25-209.25-2
FB3-P	Fladstrand	6975716	parasite eggs	CGG-3-006353	Cla6-104.25-209.25-3
FB3	Fladstrand	3563685	sediment	CGG-3-006353	Cla6-104.25-209.25-3
FC-P	Fladstrand	15142728	parasite eggs	CGG-3-006354	Cla6-100-201.0
FC	Fladstrand	36232568	sediment	CGG-3-006354	Cla6-100-201.0
FD-P	Fladstrand	18840140	parasite eggs	CGG-3-006355	Cla6-104.0-207.0
FD	Fladstrand	18042348	sediment	CGG-3-006355	Cla6-104.0-207.0
S1-P	Norse A	8927177	parasite eggs	-	V51-1
S1	Norse A	34129091	sediment	-	V51-1
S2	Norse A	31622972	sediment	-	V51-2
S3	Norse A	37730534	sediment	-	V51-3
S4-P	Norse B	11307868	parasite eggs	-	V51-4
S4	Norse B	35154452	sediment	-	V51-4
S5	Norse B	24152070	sediment	-	V51-5
S5-P	Norse B	8927051	parasite eggs	-	V51-5
S6-P	Norse C	9783576	parasite eggs	-	V51-6
S6	Norse C	14332072	sediment	-	V51-6
S7	Norse C	31324404	sediment	-	V51-7
S8-P	Norse D	10275581	parasite eggs	-	V51-8
S8	Norse D	34745133	sediment	-	V51-8
S9	Norse D	21549472	sediment	-	V51-9
S10-P	Norse D	18481341	parasite eggs	-	V51-10
S10	Norse D	22174720	sediment	-	V51-10
QB1-P	Dorset	49724763	parasite eggs	CGG-3-002130	DC-1
QB1	Dorset	28263734	sediment	CGG-3-002130	DC-1
QB2-P	Dorset	24790458	parasite eggs	CGG-3-002134	DC-5
QB2	Dorset	27216862	sediment	CGG-3-002134	DC-5
QB3-P	Peat B	25059461	parasite eggs	CGG-3-002136	DC-7
QB3	Peat B	33287966	sediment	CGG-3-002136	DC-7
QB4-P	Peat B	27305476	parasite eggs	CGG-3-002137	DC-8
QB4	Peat B	28912824	sediment	CGG-3-002137	DC-8
QB5	Peat B	37008610	sediment	CGG-3-002140	DC-11
QA1-P	Peat A	6071890	parasite eggs	CGG-3-002116	A1-28-8
QA2-P	Peat A	5599888	parasite eggs	CGG-3-002117	A1-28-9
QA2	Peat A	23252065	sediment	CGG-3-002117	A1-28-9
QA3	Peat A	24496201	sediment	CGG-3-002116	A1-28-10
QA4-P	Late Saqqaq	28726008	parasite eggs	CGG-3-002120	A1-28-12
QA4	Late Saqqaq	40545243	sediment	CGG-3-002120	A1-28-12
QA5-P	Late Saqqaq	34138026	parasite eggs	CGG-3-002121	A1-28-13
QA5	Late Saqqaq	38776479	sediment	CGG-3-002121	A1-28-13
QA6-PI	Middle Saqqaq	21149076	parasite eggs	CGG-3-002123	A1-28-15
QA6-PII	Middle Saqqaq	24908418	parasite eggs	CGG-3-002123	A1-28-15
QA6	Middle Saqqaq	365686030	sediment	CGG-3-002123	A1-28-15
QA7-P	Early Saqqaq	22336072	parasite eggs	CGG-3-002125	A1-28-17
QA7	Early Saqqaq	32655696	sediment	CGG-3-002125	A1-28-17
QA8-PI	Early Saqqaq	63211742	parasite eggs	CGG-3-002126	A1-28-18
QA8-PII	Early Saqqaq	46273685	parasite eggs	CGG-3-002126	A1-28-18
QA8	Early Saqqaq	94865064	sediment	CGG-3-002126	A1-28-18
QT1-P	Qt	12402646	parasite eggs	CGG-3-001018	Qt09-7-hul-top
QT2-P	Qt	9022699	parasite eggs	CGG-3-001019	Qt09-7-hul-bund
QT3-P	Qt	8749779	parasite eggs	CGG-3-001020	Qt09-8
QT3	Qt	222607436	sediment	CGG-3-001020	Qt09-8
QT4	Qt	44527697	sediment	CGG-3-001020	Qt09-8

Supplementary Table 9 | Library preparation details.

AAR	sample name	Sample type	mass (mg)	cm below surface	14C Age (BP)	Calibrated age
22808	V51-3	plant (E.nigrum)	2.5	15	668 +/- 25	68.2% probability<br>1283AD (38.9%) 1302AD<br>1367AD (29.3%) 1383AD<br>95.4% probability<br>1277AD (52.8%) 1316AD<br>1355AD (42.6%) 1390AD
22809	V51-5	plant (unidentified)	1.6	29	719 +/- 25	68.2% probability<br>1269AD (68.2%) 1287AD<br>95.4% probability<br>1256AD (93.5%) 1300AD<br>1370AD (1.9%) 1380AD
22810	V51-7	plant (unidentified)	4.2	44	556 +/- 30	68.2% probability<br>1324AD (29.7%) 1345AD<br>1393AD (38.5%) 1417AD<br>95.4% probability<br>1310AD (46.3%) 1361AD<br>1386AD (49.1%) 1430AD
22811	V51-10	wood (unidentified)	25.9	64	701 +/- 28	68.2% probability<br>1271AD (68.2%) 1297AD<br>95.4% probability<br>1262AD (80.5%) 1306AD<br>1363AD (14.9%) 1385AD
21594	V51-10	bone	386	64	625 +/- 25	68.2% probability<br>1298AD (26.1%) 1320AD<br>1350AD (42.1%) 1391AD<br>95.4% probability<br>1290AD (95.4%) 1398AD

Supplementary Table 10 | ^{14}C Datings from samples at Sandnes.

	Fladstrand/QT			Qajaa A/B		Qajaa profile A				Qajaa profile B			Sandnes						
Sample name	F-EB	F-LB	F-P-EB	Q-P-EB	Q-P-LB	Q-PB	QA-EB1	QA-EB2	QA-LB	QB-EB1	QB-EB2	QB-LB	S-EB1	S-EB2	S-EB3	S-EB4	S-LB	S-P-EB	S-P-LB
Bos	-	-	-	6	3	-	-	-	2	-	-	-	-	-	-	-	-	9	5
Canis lupus	-	-	-	-	-	-	-	-	-	-	-	-	-	-	-	-	-	3	-
Homo	-	-	-	-	-	-	-	-	-	-	-	-	-	-	-	-	-	5	-
Homo sapiens	-	-	-	2	-	-	-	-	2	-	-	-	-	-	3	-	3	11	-
Sus	-	-	-	-	-	-	4	-	-	-	-	-	-	-	-	-	-	2	-
Sus scrofa	-	-	-	-	-	-	2	-	-	-	-	-	-	-	-	-	-	2	-
Sus scrofa taivanus	-	-	-	-	-	-	-	-	-	-	-	-	-	-	-	-	-	2	-
Phasianidae	-	-	-	-	-	-	-	-	-	-	-	-	-	-	-	-	-	3	-
Gallus	-	-	-	-	-	-	-	-	-	-	-	-	-	-	-	-	-	4	-
Gallus gallus	-	-	-	-	-	-	-	-	-	-	-	-	-	-	-	-	-	4	-
Meleagris gallopavo	-	-	-	2	-	-	-	-	-	-	-	-	-	-	-	-	-	13	-
Gonorynchus greyi	-	-	-	-	-	-	-	-	-	-	2	-	-	-	-	-	-	-	-
Leontopithecus rosalia	-	-	6	14	-	-	34	8	29	14	3	10	5	-	-	-	-	102	2

Supplementary Table 11 | Control libraries. Vertbrate DNA reads from control samples. Samples denoted x-P-x represent parasite libraries. EB: Extraction blank, LB: Library blank, PB: Index PCR blank. Read counts below two are not shown.

group	pp. reads	vtbr. reads	vtbr. reads /Mread	plant reads	plant reads /Mread
Fladstrand	258667294	185	0.7	142654	551.5
Norse A	112409774	0	0	33805	300.7
Norse B	79541441	198	2.5	40167	505
Norse C	55440052	153	2.8	23317	420.6
Norse D	107226247	251	2.3	36891	344
Dorset	129995817	161	1.2	18692	143.8
Peat B	151574337	10	0.1	3291	21.7
Peat A	59420044	5	0.1	3546	59.7
Late Saqqaq	142185756	197	1.4	7180	50.5
Middle Saqqaq	411743524	9150	22.2	150694	366
Early Saqqaq	259342259	746	2.9	109575	422.5
QT	297310257	5310	17.9	146050	491.2

Supplementary Table 12 | DNA details of grouped samples

Supplementary Note 1 - Methodical biases and contamination

To monitor lab contamination during extraction, library building and indexing, a collection of control reactions were included in each batch of sample preparation. From examination of these control samples we detect a low background contamination of DNA from human, cow and chicken (Supplementary table 11), all of which are well known lab contaminants[11,12]. Based on this, DNA from the order of *Primates* and *Phasianidae* was identified as contamination and discarded. While sheep and goat have been identified previously as common contaminants, we do not identify these species in either peat or control samples. The background contamination from cattle DNA represents 1-5 reads per group and is detected in peat layers without evidence of cultural remains as well as Inuit layers (marked with * in Supplementary Table 1). At Sandnes, however, a strong and consistent signal from Cattle of ~30x the background contamination (82 – 117 reads per group) suggests additional endogenous DNA from the Norse cattle in these layers. As a result DNA from *Bos* was included in the dataset (Supplementary Table 2), while reads assumed to represent *Bos* contamination were omitted in Figure 2. Furthermore, in the extraction blank from the helminth library preparation of samples from Sandnes (S-P-EB) we detect 3 Canis lupus reads. While we identify Canis lupus reads in samples from both the helminth and the sediment library preparation batch, this could indicate that Canis lupus reads at Sandnes represent a contamination. Hence, Canis reads from Sandnes were marked as potential contamination and omitted in Figure 2. Reassuringly, no DNA from marine mammals where detected in any of the control reactions.

Apart from the background contamination, a low level of false positives is observed from close phylogenetic relatives to the expected species, such as the identification of *Phoca largha* and *Phoca fasciata* in libraries with high concentrations of harp seal DNA or the identification of *Pusa sibirica* and *Pusa caspica* in libraries with high concentrations of ringed seal DNA (Supplementary table 2). Such false positives are presumed to be an effect of DNA damage or sequencing errors causing a read to resemble a close relative to the true species. This hypothesis was tested by trimming all reads from each end with two trim sizes: 2bp and 5bp. Using these trimming settings we detect a reduction in reads assigned to the 4 expected false positives *Phoca largha, Pusa sibirica, Pusa caspica* and *Phoca fasciata* from 68 reads in total to 44 and 24 reads in total for 2bp and 5bp trimming from each end, respectively. This suggests that a large fraction of these assignments can be explained by 5' C to T misincorporations. However, with these trimming settings we loose valuable data, as a fraction of the asigned reads – 7.1% and 27.2% of all vertebrate reads for 2bp and 5bp, respectively – drops below the size threshold. Based on these results, we have decided to retain as much data as possible by not applying any trimming.

In addition to false positives from close phylogenetic relatives, we detect false positives presumed to be a result of closest match assignments where the true species of origin is absent from the database. This is illustrated by the identification of *Larus dominicanus* at Qajaa. Both *Larus hyperboreus* and *Larus glaucoides* have previously been identified at Qajaa[13], however, these species are absent from the mitochondrial database. Accordingly, the identification of *Larus dominicanus* is assumed to represent a closest match assignment (Supplementary table 3). Lastly, we detect DNA from *Turdus merula* in allmost all groups. These reads map exclusively to a 74bp region of the *Turdus merula* mitochondrial genome, which has high sequence similarity to bacterial DNA. Hence this identification most likely represents a false positive.

In the analysis of plant content in the sediments, we identify DNA from the family *Juglandaceae* which is not native to Greenland. Considering the low resolution in the taxonomic identification of plants and the co-occurrence of DNA from *Juglandaceae* and *Betluceae*, these reads could very well represent authentic DNA from the birch family.

Another potential bias comes from the higher coverage of GC-rich areas obtained from next generation sequencing data. To test whether this bias had any effect on the species identified here, we compared the GC content of each sequence in the mitochondrial database with the GC-content of the mitochondrial sequences from the species identified by the LCA algorithm. With a mean GC-content of 38.1% (SD: 8.5%) in the full mitochondrial database and 37.9% (SD: 8.1%) among the mitochondrial genomes from species identified here, we conclude that the GC bias of next-generation sequencing did not affect our results. Lastly, a bias could be introduced from DNA leaching between sediment layers. However, the absence of mammal DNA in the peat samples confirms that DNA leaching is not a problem in this experiment. Even though a substantial amount of vertebrate reads are identified in the Dorset layer at Qajaa, no signal from vertebrate species are detected in the peat layer below.

Supplementary Note 2 - Authenticity of results

While a few studies have been published[1–4], the use of shotgun sequencing to characterize compositions of higher eukaryotes remains largely untested, and thus results should be scrutinized to confirm data authenticity. Several lines of evidence point towards the validity of the data presented in this study. First, strict precautions were taken, both at the experimental and analytical stages of the study to ensure data authenticity. Sampling were carried out wearing gloves and facemask, and sample extraction and library building were carried out in dedicated ancient DNA facilities following strict aDNA guidelines[56]. At the analytical stages of the study, data authenticity is ensured by the use of a database containing all available mitochondrial genomes within *Metazoa,* as opposed to a small, curated database containing only species expected to be present in a given sample[7]. Furthermore, instead of maximizing the output by mapping to all sequencing data available[1], we limit the database to contain only mitochondrial DNA, assuring that each species in the database is represented by similar quantities of DNA data. Lastly, we apply strict filtering to remove duplicate reads and DNA of low complexity or poor quality (see Methods). As a result, we are able to reliably identify the majority of mammal species present in the ancient refuse, even though no *a priori* assumptions of the expected findings were made (see Fig. 3). A second factor confirming the authenticity of the data is the presence of the unambiguous DNA damage patterns associated with ancient DNA, presented in Fig. 2 and Supplementary Figures 3-5. Illustrating the time dependent nature of DNA damage accumulation, these post-mortem modifications are more pronounced in the oldest Saqqaq layers, while they are entirely absent in the young deposits at Sandnes and Fladstrand[8]. Furthermore, read coverage across the entire mitochondrial genomes of harp seal, bowhead whale and *Taenia hydatigena*, demonstrated in Supplementary Figures 3-5, serves as an additional proof of data validity, since laboratory contamination from PCR fragments are expected to map exclusively to small regions of the reference[9]. Lastly, the congruency of plants identified with *trn*L metabarcoding, shotgun metagenomics and previous macrofossil and pollen analyses at Sandnes[10] provides compelling evidence for data authenticity (Supplementary Figure 2).

Supplementary Note 3 – Inferring biomass from DNA profiles

The sedaDNA approach applied here provides an excellent means to investigate the taxonomic distribution across a variety of taxa based on a few grams of sediment. As

demonstrated in Figure 3, there is a good correlation between the DNA read counts and the expected biomass for harp seal, ringed seal, birds and fox at Qeqertasussuk. However, as discussed in the manuscript, the DNA distribution might not always reflect the biomass of the different species, as, e.g. defacation and urine might inflate the DNA record for domesticate species. Hence, when analysing sedaDNA results, the DNA sources should be carefully considered and, if available, the DNA data should be correlated with osteological evidence. In this study, the identification of hardened blubber oil within the sediment at Qeqertasussuk (Morten Meldgaard, personal communication) together with the absence of associated cetacean bones, suggests that the main source of bowhead whale DNA at Qeqertasussuk is blubber and meat. Alternative sources of DNA from marine mammals in this study could arise from the proccessing and usage of blubber. Blubber from seals or whales were used as fuel in lamps[14]; If such lamps were emptied onto the midden, the DNA signal from marine mammals could have been inflated. Similarly, the wastewater from boiling of skin and blubber in order to retrieve oil could have been discarded at the midden. However, the contribution of such alternative sources of DNA is unlikely to be significant as the blubber was heated, causing the DNA to be heavily damaged. In summary, based on the presented evidence, it cannot be conclusively shown that the biomass for bowhead whale can be inferred directly from the DNA read counts, as is the case for harp seal. However, it can safely be concluded that the level of bowhead whale exploitation at Qerqertasussuk and Qajaa, by far exceeds what has been estimated from the bone record previously.

Supplementary Methods

DNA Metabarcoding. The *trn*L p6 loop of plant chloroplasts was amplified using the primers *trn*L-*g* (5'-GGGCAATCCTGAGCCAA) and *trn*L-*h* (5'-CCATTGAGTCTCTGCACCTATC) described in[7], each tagged with a unique 6 nucleotide 5' identifier to distinguish sequences from different samples[15]. *trn*L sequences were generated from 16 extracts from Sandnes, every sample represented by at least one PCR reaction. For samples S2 -3, -4, -5, -6 and -7, *trn*L amplicons were generated in duplicates. To enhance the PCR reaction, DNA extracts were subjected to a secondary inhibitor removal step with the PowerClean® Pro Clean-Up Kit (MO-BIO). Depending on the DNA concentration, 1 to 5 µL purified extract was added to each 25µL reaction. PCR amplifications were carried out with 0.2µL Omni Klentaq DNA polymerase (DNA Polymerase Technology, Inc.) for 55 cycles in a reaction mixture with 2.5µL buffer, 12.5 µL PCR Enhancer Cocktail P (DNA Polymerase Technology, Inc.), 10mM dNTP and 1mM of each primer. The following PCR conditions were applied: 94°C for 4 min, 55 cycles of: 94°C for 30 seconds, 57°C for 30 seconds and 68°C for 60 seconds, followed by a final elongation phase at 68°C for 7 minutes. After purification with MinElute columns, PCR products were visualized on an agarose gel (2%) and a 2200 Tapestation (Agilent) using the D1000 screen tape assay. Lastly, PCR products were pooled in equimolar amounts, based on DNA concentrations measured on a Qubit fluorometer (Life Technologies) and prepared for sequencing using the NEBNext DNA Library Prep Master Mix for 454 (E6070) as described in Methods.

Sequence Analysis of Plant Barcodes. Data from *trn*L amplicons were demultiplexed and trimmed with Novobarcode and AdapterRemoval as described in Methods, only retaining collapsed reads. Followingly, amplicon reads were assigned to their corresponding samples based on primer tags using the ngsfilter program from the OBITOOLS package (http://www.grenoble.prabi.fr/trac/OBITools). Only sequences with two tags showing a complete match and primers with maximum 2 mismatches were considered. Next, reads were dereplicated with obiuniq and denoised with obigrep, discarding sequences shorter than 10bp or

represented by fewer than 2 reads. The obiclean program[16] was then applied to cluster variants of the same sequence as a result of amplification or sequencing errors, linking two sequences if the count of the rare sequence was less than 5% of the count of the abundant sequence. Subsequently, the ecoTag[17] program was applied to assign taxa to each amplicon, using a custom made database of *trn*L sequences from plant species of Greenland described in Bocher[18]. Finally, taxa represented by less than five reads or assigned at a taxonomic resolution above family level were discarded.

Phylogenetic analyses and NMDS plots. Consensus sequences were called using ANGSD[19] (0.911) and phylogenetic trees were constructed with MrBayes (3.2.6)[20], based on best substitution models identified by jModelTest (2.1.7) [21]: GTR+I+G for whale and helminth phylogeny and GTR+G for harp seal phylogeny. For each tree, four runs of four MCMC chains were run for 5,000,000 iterations sampling every 1,000 generations. Majority rule consensus trees were constructed using a burnin of 25% (`sumt Contype = Allcompat relburnin =yes burninfrac = 0.25`) and visualized with FigTree (1.4.2) (http://beast.bio.ed.ac.uk/figtree).

Variations within vertebrate and plant taxa were visualized with non-metric multidimensional scaling (NMDS) plots based on hellinger transformed bray-curtis distance calculations, using the R packages vegan (https://cran.r-project.org/web/packages/vegan/index.html) and MASS (https://cran.r-project.org/web/packages/MASS/index.html). NMDS plots were based on reads assigned within *Vertebrata* and *Viridiplantae*, respectively. For vertebrates, plots were generated from read counts of the 42 vertebrate taxa identified in Supplementary Table 2 and 3. Samples from peat layers were excluded, since no vertebrates reads were identified in these libraries. For plants, the plotting is based on plant families represented by more than 50 reads across the data set.

Supplementary References

1. Smith, O. *et al.* Sedimentary DNA from a submerged site reveals wheat in the British Isles 8000 years ago. **2,** (2014).
2. Srivathsan, A., Sha, J. C. M., Vogler, A. P. & Meier, R. Comparing the effectiveness of metagenomics and metabarcoding for diet analysis of a leaf-feeding monkey (Pygathrix nemaeus). *Mol. Ecol. Resour.* 250–261 (2014). doi:10.1111/1755-0998.12302
3. Bon, C. *et al.* Coprolites as a source of information on the genome and diet of the cave hyena. *Proc. R. Soc. B Biol. Sci.* **279,** 2825–2830 (2012).
4. Paula, D. P. *et al.* Detection and decay rates of prey and prey symbionts in the gut of a predator through metagenomics. *Mol. Ecol. Resour.* 880–892 (2015). doi:10.1111/1755-0998.12364
5. Willerslev, E. & Cooper, A. Ancient DNA. *Proc. Biol. Sci.* **272,** 3–16 (2005).
6. Gilbert, M. T. P., Bandelt, H. J., Hofreiter, M. & Barnes, I. Assessing ancient DNA studies. *Trends Ecol. Evol.* **20,** 541–544 (2005).
7. Taberlet, P. *et al.* Power and limitations of the chloroplast trnL (UAA) intron for plant DNA barcoding. *Nucleic Acids Res.* **35,** (2007).
8. Sawyer, S., Krause, J., Guschanski, K., Savolainen, V. & Pääbo, S. Temporal patterns of nucleotide misincorporations and DNA fragmentation in ancient DNA. *PLoS One* **7,** (2012).
9. Kjartansdóttir, K. R. *et al.* Traces of ATCV-1 associated with laboratory component contamination. *Proc. Natl. Acad. Sci.* **112,** E925–E926 (2015).

10. Fredskild, B. & Humle, L. Plant remains from the Norse farm Sandnes in the Western Settlement, Greenland. *Acta Boreal.* **1,** 69–81 (1991).
11. Leonard, J. A. *et al.* Animal DNA in PCR reagents plagues ancient DNA research. *J. Archaeol. Sci.* **34,** 1361–1366 (2007).
12. Haile, J. *et al.* Ancient DNA reveals late survival of mammoth and horse in interior Alaska. *Proc. Natl. Acad. Sci. U. S. A.* **106,** 22352–22357 (2009).
13. Mohl, J. Dog Remains from a Paleoeskimo Settlement in West Greenland. *Arctic Anthropol.* **23,** 81–89 (1986).
14. Grønnow, B., Appelt, M. & Odgaard, U. in *Northern Worlds - landscapes, interactions and dynamics* 403–422 (2014).
15. Binladen, J. *et al.* The use of coded PCR primers enables high-throughput sequencing of multiple homolog amplification products by 454 parallel sequencing. *PLoS One* **2,** 1–9 (2007).
16. Bellemain, E. *et al.* Fungal palaeodiversity revealed using high-throughput metabarcoding of ancient DNA from arctic permafrost. *Environ. Microbiol.* **15,** 1176–1189 (2013).
17. Yoccoz, N. G. *et al.* DNA from soil mirrors plant taxonomic and growth form diversity. *Mol. Ecol.* **21,** 3647–3655 (2012).
18. Bocher, T. W., Fredskild, B., Holmen, K. & Jakobsen, K. *Grønlands Flora*. (P. Haase & Søns Forlag, 1978).
19. Korneliussen, T. S., Albrechtsen, A. & Nielsen, R. ANGSD: Analysis of Next Generation Sequencing Data. *BMC Bioinformatics* **15,** 356 (2014).
20. Ronquist, F. & Huelsenbeck, J. P. MrBayes 3: Bayesian phylogenetic inference under mixed models. *Bioinformatics* **19,** 1572–1574 (2003).
21. Darriba, D., Taboada, G. L., Doallo, R. & Posada, D. jModelTest 2: more models, new heuristics and parallel computing. *Nat. Methods* **9,** 772–772 (2012).
22. Meldgaard, M. Ancient Harp Seal Hunters of Disko Bay. *Meddelelser om Grønl. Man Soc.* (2004).
23. Carr, S. M., Duggan, A. T., Stenson, G. B. & Marshall, H. D. Quantitative Phylogenomics of Within-Species Mitogenome Variation: Monte Carlo and Non-Parametric Analysis of Phylogeographic Structure among Discrete Transatlantic Breeding Areas of Harp Seals (Pagophilus groenlandicus). *PLoS One* **10,** e0134207 (2015).
